# Health and the 2008 Economic Recession: Evidence from the United Kingdom

**DOI:** 10.1371/journal.pone.0056674

**Published:** 2013-02-20

**Authors:** Thomas Astell-Burt, Xiaoqi Feng

**Affiliations:** 1 School of Science and Health, University of Western Sydney, Sydney, Australia; 2 Centre for Health Research, School of Medicine, University of Western Sydney, Sydney, Australia; 3 School of Geography and Geosciences, University of St Andrews, St Andrews, United Kingdom; Boston Children's Hospital, United States of America

## Abstract

**Introduction:**

The economic recession which began in 2008 has resulted in a substantial increase in unemployment across many countries, including the United Kingdom. Strong association between unemployment and poor health status among individuals is widely recognised. We investigated whether the prevalence of poor health at a population level increased concurrent to the rise in unemployment during the economic recession, and whether the impact on health varied by geographical and socioeconomic circumstances.

**Method:**

Health, demographic and socioeconomic measures on 1.36 million survey responses aged 16–64 were extracted from the Quarterly Labour Force Survey of the United Kingdom, collected every three months, from January 2006 to December 2010. The likelihood of self-reporting poor health status and specific types of health problems (depression, mental illness, cardiovascular and respiratory) across time were estimated separately using logistic regression. Explanatory variables included economic status (International Labour Organization definition), occupational class, age, gender, country of birth, ethnicity, educational qualifications, couple status, household tenure, number of dependents, and geographical region.

**Results:**

Unemployment (age-gender adjusted) rose from 4.5% in January 2008 to 7.1% by September 2009. The reporting of poor health status increased from 25.7% in July 2009 to 29.5% by December 2010. Similar increases were found for cardiovascular and respiratory health problems; not depression or mental illness. The prevalence of poor health status among the unemployed decreased from 28.8% in July 2008, to 24.9% by March 2009; but this was followed by an increase in poor health experienced across all regions and by all socioeconomic groups, including those who remained employed, regardless of their occupational class.

**Interpretation:**

Although our study found no exacerbation of pre-recession health inequalities, the rise in poor health status not only for the unemployed, but also among people who remained employed, regardless of their occupational class, justifies concern voiced among many public health commentators.

## Introduction

Given the association between unemployment and poor health [1], [2] the rapid increase in layoffs from 2008 onwards in the United Kingdom (UK) and United States (US) has raised alarm bells among many public health commentators [3], [4], [5], [6], [7]. While evidence of an effect of previous recessions on health in the US [8], [9], [10], [11], Europe [12], [13], [14], [15], [16], [17], [18] and Asia [19], [20] is equivocal, preliminary findings from the most recent crisis appear to confirm fears of a rise in the number of suicides [17], [18].

It is unlikely that the 2008 economic recession has influenced everyone to the same extent. The unemployed tend to have poorer health than the employed [1], [2], but people in routine/manual labour occupations are also known to suffer more health problems than their peers in managerial/professional occupations [21]. It was the UK's manufacturing sector which experienced the most substantial fall in employment between 2008 and 2009 [22]. Health problems stemming from job loss and increased job insecurity [9], [10], [23], [24] are therefore likely to have been borne disproportionately among the unemployed and those employed in routine/manual labour occupations. As industries at risk are spatially patterned, some areas of the UK were likely to be more vulnerable to the effects of the recession than others, potentially widening geographical inequalities in health [18].

Using a large survey repeated every three months from January 2006 to December 2010, we investigated to what extent poor health status, and inequalities thereof according to geographical and socioeconomic circumstance, rose during the 2008 economic recession.

## Methods

### Design

Research in this field, according to Catalano and colleagues [10], can be classified into two groups characterised by design: *i)* risk factor studies; and *ii)* net effect studies. Risk factor studies are those which attempt to identify the effects of unfavourable economic circumstances among individuals, such as financial insecurity or involuntary job loss, on their health and behaviours. In contrast, net effect studies provide an insight into the sum of the economic effects on population health by using groups or geographical areas as the analytical units, and estimating temporal change in association between economic indicators (such as the rate of unemployment) and prevalence or incidence rates.

In this paper, we used individual-level data on health status, demographic and socioeconomic circumstances extracted from the UK Quarterly Labour Force Survey (QLFS) [25] to conduct research most akin to the ‘net effects’ study design. The QLFS is among the largest regular social surveys in the UK, collecting cross-sectional information every three months (‘quarters’) from over 100,000 people living in private households across all areas of England, Scotland, Wales and Northern Ireland. To capture circumstances before, during and since the economic recession which began in 2008, we pooled 60 months (20 quarters) of the QLFS from January 2006 to December 2010 to create a dataset of 2.38 million survey responses (1.15 million men, 1.23 million women). Our focus was on the health of the working-age population, so we kept all women aged 16 y–59 y and men aged 16 y–64 y in our data (reflecting gender differences in the national retirement age). This left a sample of 1.36 million survey responses, approximately 57% of the original dataset surveyed across 20 quarters from 2006 to 2010. This very large sample size and high frequency of update were key reasons for using the QLFS in our study, in comparison to alternative sources of data which are less frequent (e.g. the decennial UK Census) or have far smaller sample sizes (e.g. British Household Panel Survey). A narrow risk factor study (e.g. to test the effect of job loss on health) was clearly possible with the QLFS, however, our main objective was to use this rich source of individual-level data to estimate the net effects of the economic recession on population health over time, across regions, and between selected groups.

### Measures

Health status in the QLFS was self-rated. The primary outcome variable was health status measured using responses to the question “*Do you have any health problems or disabilities that you expect will last for more than a year?*” 95% of all survey responses of working age in the QLFS indicated a response (yes or no). Non-responders were not asked this question on health status as they were too ill or distressed to continue with the survey; we omitted these survey responses from the analysis.

In secondary analyses, we investigated specific types of health problems reported in the QLFS that could have been influenced by the 2008 economic recession. In the interests of brevity, we assigned each group a name. The exact wording from the QLFS is given in parentheses.

- Depression (“Depression, bad nerves or anxiety”);
- Mental illness (“Mental illness, or suffer from phobia, panics or other nervous disorders”);
- Cardiovascular (“Heart, blood pressure or blood circulation problems”);
- Respiratory (“Chest or breathing problems, asthma, bronchitis”).

Individual economic status (employed, unemployed, and economically inactive – including early retirees, students, the long-term sick, and homemakers) was measured according to the International Labour Organization (ILO) definition [26]. Among those who were employed, their occupational class was identified using the UK National Statistics Socio-Economic Classification (NS-SEC) [27]. The NS-SEC groups occupations into classes to reflect working relations between employers and employees, the salaried and the temporary labour force, and the manual and non-manual workers. All occupations in the QLFS were classified into three recognized NS-SEC classes: ‘routine/manual’; ‘intermediate’; or ‘professional/managerial’.

We also utilised a number of conventional demographic measures which were surveyed in each quarter, including age, gender, country of birth, ethnicity (White, Mixed, Indian, Pakistani, Bangladeshi, Chinese, Other Asian, Black Caribbean or Black African, Other), educational qualifications (none, GCSE, A-Level, Degree, Other), couple status (married or living with partner, single never married, separated/divorced/widowed), household tenure (owned outright/with mortgage, rented, rent free, part rent/part mortgage), number of dependents (0 to 4+), and geographical region (n = 20).

### Statistical analysis

To test whether overall levels of health worsened during the 2008 economic recession, we first assessed the prevalence of each health status across all other characteristics of the study sample and each of the 20 quarters using descriptive statistics. Association between health and each explanatory variable was investigated using logit regression. Coefficients were exponentiated to odds ratios (OR), indicating the likelihood of a person reporting a health problem compared to the likelihood of not reporting a health problem.

For assessing the extent to which health varied across time, we used an explanatory variable containing 20 categories. Each category represented a single quarter (i.e. three months of data). We fitted logit regression models of each outcome variable, adjusted for this categorical variable denoting time, with the reference category set as quarter 1 (January to March 2006). An advantage of adopting this categorical approach was that we did not impose any presupposition of the distribution of health status across time. This approach was more favourable in comparison to using a combination of linear and polynomial functions of time (e.g. square and cubic functions), which were found to have a smoothing effect, resulting in underestimation of rapid changes in health occurring from one quarter to the next.

We constructed multivariate models in several steps. First, our models were adjusted by age group and gender. An interaction term between age and gender was fitted to account for differences in health between males and females at different ages. Second, we added dummy variables denoting economic status, occupational class, and geographic region into the models separately, and then simultaneously. Third, all other explanatory variables were added sequentially to the models. For each model, 95% confidence intervals (95% CI) were used to assess whether change in the unemployment rate and prevalence of health problems between each quarter was statistically significant (p<0.05).

With a view towards addressing whether changes in each of the outcome variables coincided with the 2008 economic recession, we fitted an age, gender and time adjusted logistic regression model of unemployment (vs. employment) separately. Only economically active people were included in these models, as these people were either in work or actively looking for jobs, in line with the ILO definition [26]. Indirect comparisons between trajectories in unemployment and health status were made in this way, rather than adjusting for a regional measure of unemployment, as our use of a fixed effect for region precluded the analysis of any explanatory variables at this level. However, individual economic status was included in the health models. Indirect comparisons also helped us to avoid imposing any assumptions regarding the length of time required for the economic recession to have observable influences on population health.

To investigate influence on health inequalities, we extended our multivariate logit regression of health status with interactions between time, economic status, occupational class, and geographic region. The model interacting time with occupational class omitted all people who were not employed. All of these models identified the extent of change in health status across time between people in different geographical and socioeconomic circumstances during the economic recession. All analyses were conducted in Stata v.12 (StataCorp, TX, USA).

## Results

Descriptive statistics of the entire sample pooled across all quarters are reported in Table 1. In brief, 28.4% of the overall sample reported poor health status. The most commonly reported health problems were cardiovascular (7.6%). 50% were married or living with a partner. 12.4% had no educational qualifications. 4.9% were unemployed.

**Table 1:** Descriptive statistics of the study sample.

N	1,361,216
	%
Poor health status	28.4
Health problems	
Depression	4.0
Mental illness	2.0
Cardiovascular	7.6
Respiratory	6.6
Gender	
Male	50.5
Female	49.6
Age	
16–25	18.8
26–35	19.9
36–45	25.1
46–55	22.5
56–64	13.8
Ethnicity	
White	90.2
Mixed ethnicity	0.8
Indian	2.1
Pakistani	1.5
Bangladeshi	0.5
Chinese	0.5
Other Asian	0.8
Black Caribbean	0.9
Black African	1.2
Other ethnic group	1.6
Country of birth	
UK/Rep. of Ireland	88.2
Overseas	11.8
Couple status	
Married, or living with partner	50.2
Single, never married	37.3
Separated/divorced/widowed	12.5
Educational qualifications	
None	12.4
GCSE	22.9
A-Level	23.0
Higher Ed, Degree or Equivalent	28.8
Other Qualifications	12.1
Economic status	
Employed	73.8
Unemployed	4.9
Economically Inactive	21.3
Occupational class	
Managerial and professional	33.9
Intermediate	17.4
Routine and manual	30.2
Never worked and long-term unemployed	18.5
Number of dependents	
0	53.5
1	20.1
2	18.3
3	6.0
4+	2.1
Household tenure	
Owned outright	19.1
Bought with mortgage or loan	51.9
Part rent, part mortgage	0.4
Rented	27.8
Rent free	0.8
Region of residence	
Tyne and Wear	2.0
Rest of North East	2.6
Greater Manchester	4.2
Merseyside	2.1
Rest of North West	4.9
South Yorkshire	2.3
West Yorkshire	4.0
Rest of Yorkshire and Humberside	2.8
East Midlands	7.6
West Midlands Metropolitan Area	3.9
Rest of West Midlands	4.6
East of England	9.2
Inner London	4.0
Outer London	6.5
South East	13.6
South West	8.2
Wales	4.8
Strathclyde	3.6
Rest of Scotland	5.0
Northern Ireland	4.1

Created by the Authors using the UK Quarterly Labour Force Survey Jan–Mar 2006 to Oct–Dec 2010.

Table 2 reports the rate of unemployment, poor health status and health problems in Jan–Mar '06. The unemployment rate was adjusted for age and gender. The rates of poor health and health problems were adjusted for all explanatory variables. The second column reports the difference in each rate between Jan–Mar '06 and '08 (i.e. change prior to the beginning of the economic recession). The third column also reports the difference in each rate by Oct–Dec '10, though in comparison to Jan–Mar '08 (i.e. change during the recession). Unemployment between Jan–Mar '06 and '08 was relatively stable at 4.5%, though it then climbed between Jan–Mar '08 and Oct–Dec '10 by 2.5 percentage points. A similar pattern of consistency pre-recession was found for the prevalence of poor health status prior to the recession, followed by a 4.7 percentage point increase between Jan–Mar '08 and Oct–Dec '10. To a much smaller extent, this pattern was also found for the prevalence of respiratory and cardiovascular health problems, though not for depression and mental illness.

**Table 2:** Prevalence of unemployment, poor health status, and health problems, before and during the UK economic recession of 2008.

	Jan–Mar '06	Jan–Mar '08	Oct–Dec '10
	%	% change	
Unemployment	4.5	+0.0	+2.5
			
Poor Health Status	25.1	−0.3	+4.7
Health Problems			
Respiratory	5.6	+0.0	+1.0
Cardiovascular	3.7	+0.2	+0.6
Depression	1.6	+0.2	+0.3
Mental Illness	0.5	+0.1	+0.1

Unemployment: Calculated according to the International Labour Organisation definition.

Jan–Mar '06: Prevalence of unemployment, poor health status, and health problems during Jan–Mar '06.

Jan–Mar '08: Percentage-point difference in prevalence between Jan–Mar '08 and Jan–Mar '06.

Oct–Dec '10: Percentage-point difference in prevalence between Oct–Dec '10 and Jan–Mar '08.

Created by the Authors using the UK Quarterly Labour Force Survey Jan–Mar 2006 to Oct–Dec 2010.

**Figure 1:**
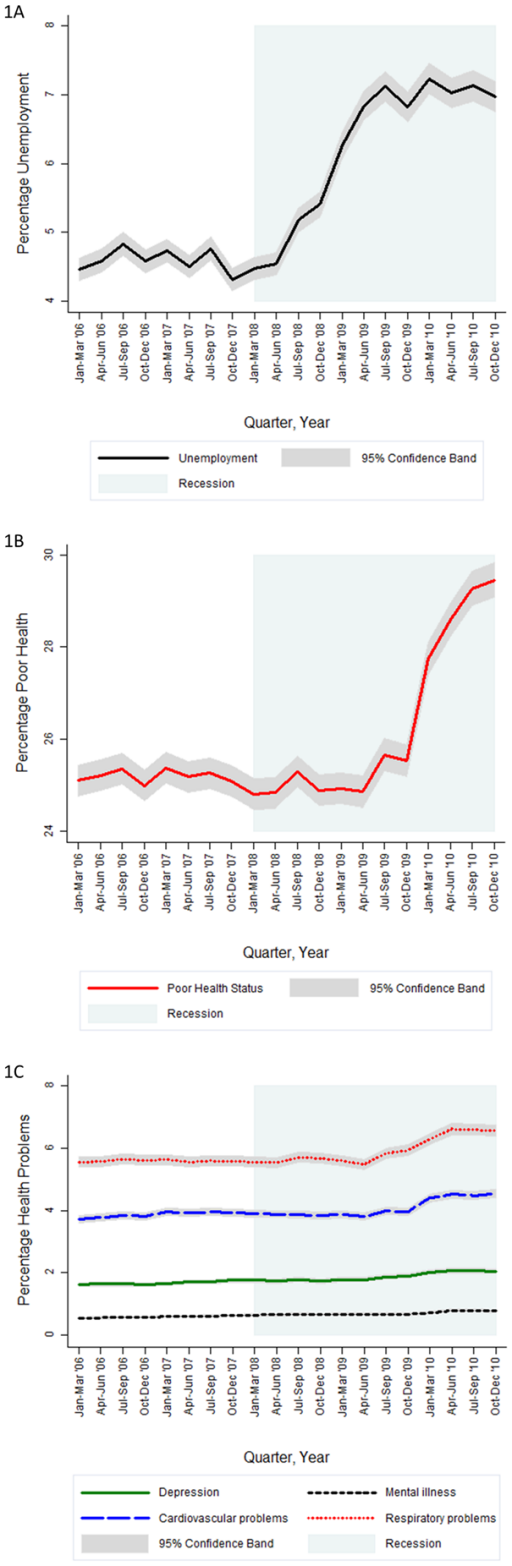
Prevalence of unemployment^1a^, poor health status^1b^ and health problems^1c^ with 95% confidence bands across time (quarterly) from January–March 2006 to October–December 2010 (inclusive). ^1a^ Unemployment is based upon the International Labour Organization (ILO) definition, using only the economically active subsample (unemployed + employed) and adjusted for quarter (categorical format), age group, gender, age group x gender. ^1b^ Poor health status is adjusted for quarter, age group, gender, age group x gender, economic status, NS-SEC occupational class, educational qualifications, couple status, household tenure, country of birth, ethnicity, number of dependents, geographical region. ^1c^ Health problems (in separate models) are adjusted for quarter, age group, gender, age group x gender, economic status, NS-SEC occupational class, educational qualifications, couple status, household tenure, country of birth, ethnicity, number of dependents, geographical region. Created by the Authors using the UK Quarterly Labour Force Survey Jan–Mar 2006 to Oct–Dec 2010.

These tables are useful for summarising change in each rate over time, but they may also obscure important information on timing of change. To elucidate this information, we modelled the risk of unemployment (age-gender adjusted), poor health status and health problems (both fully adjusted) for every quarter between Jan–Mar '06 to Oct–Dec '10 inclusive. Figure 1 illustrates the timing of change in each rate, with statistical significance denoted by the 95% confidence band. 1A shows the rapid increase in unemployment between Jan–Mar '08 and Jul–Sep '09, after which it stabilised around 7%. After full adjustment, the odds ratio (OR) of reporting poor self-rated health was 1.38 (95% confidence interval (95% CI) 1.35, 1.40) among unemployed compared to employed. In contrast, the rise in poor health status (1B) and respiratory health problems (1C) only appeared to begin from Apr–Jun '09. Cardiovascular health problems seemed to increase much later, from Oct–Dec '09 onwards. Fully-adjusted association between unemployment and each type of health problem was as follows: *i)* respiratory health problems OR: 1.20 (95% CI 1.16, 1.24); *ii)* cardiovascular health problems OR: 1.05 (95% CI 1.01, 1.09); *iii)* depression OR: 2.98 (95%CI 2.85, 3.10); and *iv)* mental illness OR: 3.18 (95% CI 2.98, 3.38). Comparing 1A with 1b and 1C, it is clear that the rise in unemployment pre-dated the increasing prevalence of poor health status and selected health problems.

For our investigation of health inequality, we focused on the measure of poor health status as it showed the most substantial increase between Jan–Mar '08 and Oct–Dec '10. To examine separate trajectories by geographical and socioeconomic circumstances, an interaction term was fitted between the time variable and each group (region, economic status, and occupational class) in separate models. As before, we initially looked at fully adjusted rates in Jan–Mar '06, along with the percentage point change by Jan–Mar' 08 and Oct–Dec '10. Table 3 reports the results of this analysis, with Jan–Mar '06 cells sorted from high to low.

**Table 3:** Prevalence of poor health status across geographic and socioeconomic circumstances, before and during the UK economic recession of 2008.

	Jan–Mar '06	Jan–Mar '08	Oct–Dec '10
	%	% change	
Region			
South Yorkshire	29.2	+0.0	+4.4
Greater Manchester	28.7	−1.8	+5.9
Tyne and Wear	28.2	−1.0	+3.3
Merseyside	27.1	−1.6	+2.3
Wales	26.9	−0.6	+6.7
Outer London	26.4	−4.3	+5.7
Rest of North East	26.4	−1.3	+7.9
East Midlands	26.1	+0.3	+3.3
Rest of Yorkshire and Humberside	25.9	−3.8	+7.4
West Midlands Metropolitan Area	25.6	+1.4	+3.4
Rest of West Midlands	25.6	+0.8	+0.5
Rest of North West	25.6	−1.4	+6.9
Strathclyde	25.5	+1.3	+1.8
Rest of Scotland	25.0	−1.7	+6.0
South West	24.7	+0.7	+5.2
South East	24.6	−0.4	+5.4
West Yorkshire	24.4	+1.9	+2.8
East of England	23.7	+0.8	+5.1
Northern Ireland	20.2	+0.8	+2.2
Inner London	19.2	+2.4	+4.3
Economic Status			
Inactive	42.2	−0.3	+3.8
Unemployed	26.8	+1.9	+1.2
Employed	21.0	−0.4	+4.9
Occupational Class			
Routine and Manual	23.1	−0.8	+4.8
Intermediate	21.0	+0.3	+4.4
Managerial and Professional	20.3	−0.4	+5.2

Jan–Mar '06: Prevalence of poor health status during that Jan–Mar '06.

Jan–Mar '08: Percentage-point difference in prevalence between Jan–Mar '08 and Jan–Mar '06.

Oct–Dec '10: Percentage-point difference in prevalence between Oct–Dec '10 and Jan–Mar '08.

Created by the Authors using the UK Quarterly Labour Force Survey Jan–Mar 2006 to Oct–Dec 2010.

Geographical inequality in the prevalence of poor health status was evident in Jan–Mar '06, with 10 percentage points separating South Yorkshire (29.2) and Inner London (19.2%). The prevalence of poor health status was higher among the unemployed (26.8%), the economically inactive (42.2%), and people in routine and manual labour occupations (23.1%). During the period leading up to the beginning of the economic recession in Jan–Mar '08, only a marginal change in prevalence occurred across these regions and groups, with notable exceptions in London and the Rest of Yorkshire and Humberside region. During the recession period, increasing prevalence of poor health status was evident across the UK. However, the increase was neither evenly distributed across all regions, nor concentrated within those which had the highest prevalence rates in Jan–Mar '06 or '08. In terms of economic status, the largest increase in prevalence was for the employed, at 4.9 percentage points. In comparison, the prevalence of poor health status only increased by 1.2 percentage points. Similarly, the managerial and professional occupations reported the greatest increase in poor health status (5.2 percentage points), although this was only marginally higher than the increase among intermediate, routine and manual occupational classes. Therefore, and perhaps counterintuitively, the relative increase in poor health status was slightly higher for the employed, and among them, those in the most favourable occupational class.

**Figure 2:**
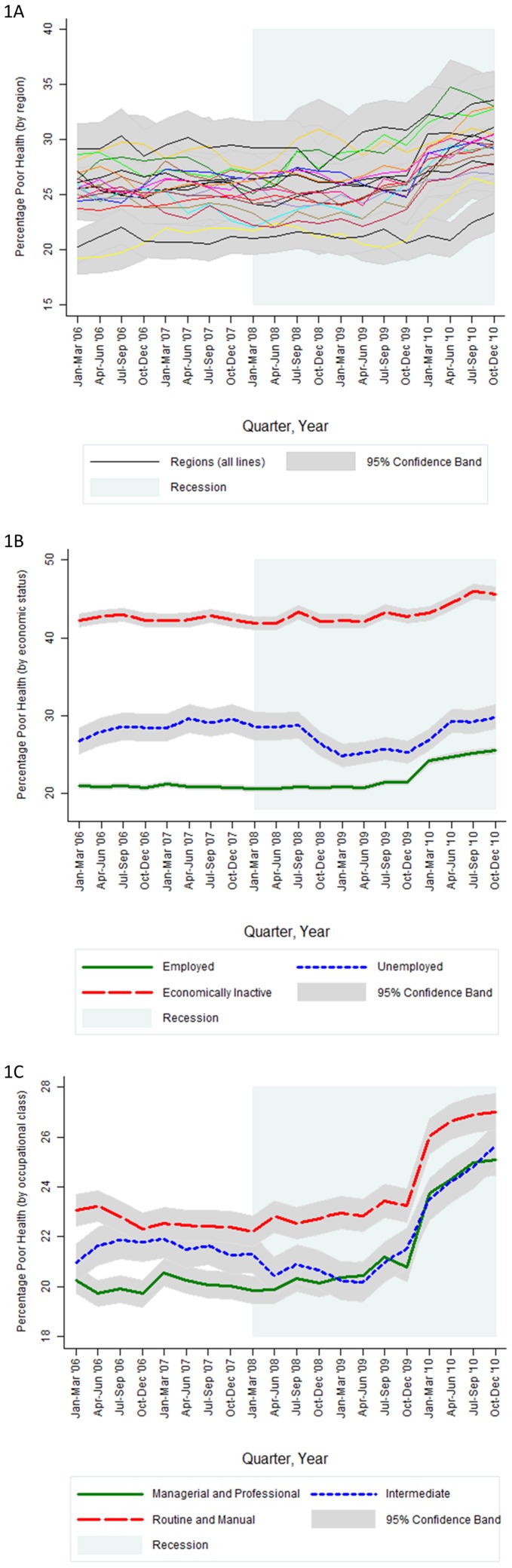
Prevalence of poor health status across time (quarterly) with 95% confidence bands, by geographic region^2a^, economic status^2b^ and occupational class^2c^ from January–March 2006 to October–December 2010 (inclusive). ^2a^ Poor health status (by region) are adjusted for quarter, geographical region, quarter x geographical region, age group, gender, age group x gender, economic status, NS-SEC occupational class, educational qualifications, couple status, household tenure, country of birth, ethnicity, number of dependents. ^2b^ Poor health status (by economic status) is adjusted for quarter, economic status, quarter x economic status age group, gender, age group x gender, NS-SEC occupational class, educational qualifications, couple status, household tenure, country of birth, ethnicity, number of dependents, geographical region. ^2c^ Poor health status (by occupational class) is adjusted for quarter, occupational class, quarter x occupational class, age group, gender, age group x gender, educational qualifications, couple status, household tenure, country of birth, ethnicity, number of dependents, geographical region. Only people who were employed were fitted within this model. Created by the Authors using the UK Quarterly Labour Force Survey Jan–Mar 2006 to Oct–Dec 2010.

Figure 2 supplements these findings with an analysis of change in prevalence across all quarters between Jan–Mar '06 and Oct–Dec '10. In general, the increase in poor health status by region (Figure 2A) tended to occur from Jul–Sep '09. In contrast, the trajectories by economic status (Figure 2B) were less straightforward. Three quarters (nine months) into the economic recession, the prevalence of poor health status began to decrease for the unemployed, from 28.8% in Jul–Sep '08 to 24.9% by Jan–Mar '09. This downward trajectory then appeared to settle until Oct–Dec '09, during which a rapid upward shift returned the prevalence of poor health status among the unemployed to a pre-recession levels. Concurrent to the rise in prevalence for the unemployed, the rate of poor health status also increased significantly for the economically inactive, and especially those who remained employed (+4 percentage points between Oct–Dec '09 and Oct–Dec '10). Among the employed, the increasing prevalence of poor health status occurred from Apr–Jun '09 for each of the occupational classes (Figure 2C).

## Discussion

### Key findings

Using a large UK-based survey repeated every three months for 20 quarters from January 2006, our study demonstrated a significant increase in the prevalence of poor health status shortly after the rise in unemployment at the start of 2008. This was reflected in similarly timed increases in cardiovascular and respiratory health problems, though not for depression and mental illness. Overall, this suggests that economic recession poses a substantively negative effect on population health.

Counterintuitively, the prevalence of poor health status among the unemployed decreased and remained lower than pre-2008 levels between Jul–Sep '08 and Oct–Dec '09, shortly after the rise in unemployment. After this period, however, the prevalence of poor health status increased significantly across the board, regardless of geographical and socioeconomic circumstances. Socioeconomic inequalities in poor health status observed prior to 2008 were not exacerbated, as slightly higher increases were experienced by people who remained employed (vs unemployed) and among those in professional and managerial occupations (vs routine and manual). Moreover, the greatest increase in poor health prevalence was not concentrated in regions with the highest pre-2008 prevalence.

### Interpretation

Previous research has suggested that adverse effects of recessions on health are most likely to occur when economic declines are rapid [4], as was the case in 2008 [22]. The substantial rise in poor health during the economic recession is an important finding. While previous research demonstrates a two-fold increase in the risk of developing long-term illness among people who become unemployed [2], controlling for economic status did not explain the increasing risk of poor health in our study. Nor did occupational class, or any of the other numerous explanatory variables we fitted in our models. This rise only appeared to occur following a period in which the prevalence of poor health among the unemployed decreased. Although this may appear counterintuitive, it is likely due to a mixing of those who were recently laid off with people who had suffered health problems associated with long-term unemployment.

The subsequent increase in poor health experienced for all groups, but particularly those who remained employed (and regardless of their occupational class), is an important finding which falls in line with previous literature. Although many people did not experience redundancy, Catalano and colleagues [10] suggest that stress associated with job-insecurity is a key mechanism for increasing poor health status during economic recession. Fear of unemployment also helps to explain why there was a time-lag between the start of the recession and the rise in poor health, as changes in feelings of job security and declining confidence in the economy are unlikely to be instantaneous across the board. Optimism among some people who felt secure at the start of the recession is likely to have diminished over time, as unemployment grew, and although it appeared to plateau, it remained high. A persistently bleak economic outlook and with hopes of a quick rebound to pre-recession prosperity looking increasingly unlikely, it would not be surprising if financial difficulties had accumulated more slowly among some people who were not instantly effected (e.g. by unemployment), to a point where it did have a significant impact on their health; even among those in more affluent circumstances. To the best of our knowledge, the only other published empirical findings (so far) of this recession on health have been reported by studies of suicide within countries of the European Union [17], [18]. They reported increasing rates of suicide immediately preceding and then during the economic recession which began in 2008. Together, these findings and ours suggest that the anticipation of potential job loss is likely to have been an important mechanism linking the 2008 economic recession with health [4], [23], [24].

Health inequalities, by economic status, occupational class or geographical region, were not exacerbated by the 2008 economic recession during the time period our study was able to observe. Despite layoffs being concentrated in sectors such as manufacturing [22], people were affected regardless of whether they were in professional/managerial or routine/manual occupations. Rising prevalence of poor health across all groups may suggest a common pathway affecting employed and unemployed people. Studies of behavioural responses to previous recessions, however, suggest the possibility for several mechanisms operating simultaneously on people in different social positions. Indeed, the effect may not always be adverse for health. For example, psychological distress [9] and suicide [17], [18] tend to increase, but mortality from motor vehicle accidents and cardiovascular disease are known to decrease [15], [16]. Alcohol consumption and tobacco smoking reportedly decrease, especially among heavy drinkers and smokers [28], [29], [30]. An increase in physical activity and decrease in bodyweight has been found among those who were previously sedentary and obese in some contexts [28], though not all [31]. Declining working hours during economic recession [22], according to some commentators [28], could free up time for investing in health-related behaviours. Meanwhile, the threat of job loss is known to have had a strong ‘disciplinary effect’ [32] not only among individuals whose sole source of income is their job, but also for those who fear social stigma, potential loss of networks and diminished status [33]. Consequently, some individuals may well have had more time to invest in healthier activities, but it is highly probable that many others will have spent extra hours worrying about how to budget loss of income and lifestyle [10], potentially increasing the risk of substance misuse as a short-term coping remedy [13]. Behavioural responses to the economic recession and their influence on health may therefore play an important role in explaining the time-lag between the start of the recession and the increase in poor health – particularly among those who remained employed. It also suggests that there may be further impacts of the recession on health which are yet to fully emerge. Therefore, an avenue for further research will be the effect of the 2008 economic recession on health-related behaviours, and how responses might have differed across the social gradient.

### Strengths and limitations

Our study benefits from the use of a very large sample of individual-level data, collected very frequently and at regular intervals before, during and since the economic recession which began in 2008. This enabled our study to detect important within-year changes in all variables, which would otherwise have been hidden by studies relying upon data collected less frequently (e.g. annually). We were able to compare the extent that the 2008 recession coincided with the reporting of a variety of health problems – some were more subject to change than others. The use of individual-level data on health, economic status, occupational class and all of our confounding variables, avoids the fallacy of inferring the experiences of individuals from combining sources of aggregated data. Furthermore, the large number of explanatory variables helped to avoid methodological problems suffered by some previous studies (e.g. omitted variables bias). We were unfortunately not able to examine the influence of neighbourhood characteristics, as this information could not be linked to the QLFS. Thus, although our study has shown that regional inequalities were not exacerbated, this does not discount the possibility that inequalities within those regions at local levels (e.g. between affluent and deprived neighbourhoods) could have widened.

Self-reported measures of health strongly correlate with mortality [34] and are also associated with important clinical information, such as inflammation in otherwise healthy adults [35], but they will not reflect the full extent of health conditions in the population and future studies might consider the use of objectively measured health status if data is available. The small effect of the recession on mental health in our analyses was unexpected, and may be due to the sensitivity of the question contained within the QLFS wherein participants were required to report health problems or disabilities that they expected to last for more than a year. This stipulation of 12 months or longer may lead to an under-reporting of mental health problems in the QLFS and it may be advisable for future surveys to include short screening instruments (e.g. [36]) to monitor trends in psychological distress at the population-level. The time-lag and rapid within-year changes identified in our study illustrate the importance of having a high frequency of follow-up surveys, but also points towards an important qualification; there may yet be further distal influences of the 2008 recession on health status, with the full effects manifesting over a much longer period of time. Longitudinal studies are warranted in the years ahead.

## Conclusion

Epidemiological studies of previous economic recessions have reported equivocal findings [10]. Although our study found no exacerbation of pre-recession health inequalities, the significant rise in poor health status not only for the unemployed, but also among people who remained employed, regardless of their occupational class, justifies concern voiced among many public health commentators [3], [4], [5], [6], [7]. Our findings suggest that economic recession poses a substantive threat to population health, and cost/benefit analyses used by decision-makers should take into account the costs of illness induced by economic policy options that threaten to increase unemployment.
